# Caesarean section – an appraisal of some predictive factors in Lagos Nigeria

**DOI:** 10.1186/1471-2393-14-217

**Published:** 2014-06-30

**Authors:** Oluwarotimi Ireti Akinola, Adetokunbo O Fabamwo, Adetokunbo O Tayo, Kabiru A Rabiu, Yusuf A Oshodi, Mercy E Alokha

**Affiliations:** 1 Department of Obstetrics and Gynaecology, Lagos State University Teaching Hospital, 1-5 Oba Akinjobi St, PO Box 53, Ikeja, Lagos, Nigeria

## Abstract

**Background:**

Several maternity units in the developing world lack facilities for caesarean section and often have to transfer patients in extremis. This case controlled study aimed to appraise predictive factors for caesarean section.

**Methods:**

One hundred and fifty two consecutive women with singleton pregnancies who had caesarean section were studied. The next parturient with normal delivery served as control. Variables such as age, parity, marital status, booking status, past obstetric history, weight, height, infant birth weight were assessed. Data obtained were analysed using SPSS 16.0 Windows package.

**Results:**

During the study period, there were 641 deliveries with 257 of them by caesarean section (40.1%).

Logistic regression analysis showed that parity, booking status, maternal height; maternal weight, birth weight, previous caesarean section and ante-partum bleeding were significant predictive factors for caesarean section while maternal age was not.

**Conclusions:**

These predictive factors should be considered in antenatal counseling to facilitate acceptance by at risk women and early referral.

## Background

Caesarean section rates have been rising progressively worldwide [1] with a wide variability amongst various countries and regions [2].

This rise has been attributed to improvement of surgical techniques, innovation, technological development [3], changes in women’s preferences, and a growing proportion of women who have previously had a caesarean section [4].

In the developed countries, caesarean section has become well established with ease and safety [3,5]. The caesarean section rate worldwide is currently stated to be between 18-35% [6,7]. Indications, such as cephalo-pelvic disproportion and fetal distress have been implicated in the rising rate of caesarean section in the tropics [6,8,9].

Despite its growing acceptance as an alternative to vaginal birth, caesarean section is not benign surgery [10], increasing the health risks for mothers and babies as well as the costs of health care compared with normal deliveries [11]. It is important to note that caesarean delivery is a major surgical procedure and peri-operative complications remain a significant source of maternal and fetal morbidity and mortality [1]. The maternal death rate following caesarean section has been quoted to be between 0.2 - 1.8% in Nigeria [12].

Various factors associated with increase in caesarean section rates have been identified. These include previous caesarean section and patients attended by a gynecologist with more than 16 years of experience [3,13], use of electronic fetal monitoring and fetal scalp blood sampling, the use of partograms, breech presentations [1], extreme ages of reproductive life, macrosomia, nulliparous and grand multiparous status [14]. Others include bleeding during pregnancy, high blood pressure, multiple pregnancy, height less than 150 cm, fetal compromise, nulliparity and presence of medical disease during pregnancy, obesity and lack of hospital antenatal care.

In developing countries, especially in sub-Saharan Africa, there is still a great aversion to caesarean section [7,8] though it may sometimes be the only means to save the life of the mother and/or foetus [2,9]. The Health system in Nigeria is stratified in a manner that majority of deliveries are initiated in centers where caesarean section cannot be offered and ambulance services are almost non existent. This accounts for the high rate of unbooked patients seen in labour at the referral hospitals who often are more likely to undergo emergency caesarean section with adverse obstetric outcomes compared to booked parturients [15-17]. Many of these patients however never access appropriate health facilities on time.

The availabilty and acceptance of caesarean section depends on early recognition of risk factors to enable caregivers antenatally counsel and refer the pregnant women to appropriate hospitals before they fall in labour [14].

Though risk factors for caesarean section have been largely defined, it was considered appropriate to evaluate these factors locally and determine their contribution to caesarean section rates in this specific population.

This study was therefore undertaken to determine the main indications and predictive factors for caesarean section at the Lagos State University Teaching Hospital.

## Methods

### Population and study design

This is a case-control study of women who had caesarean section (case) and women who had Spontaneous Vaginal Delivery (control) at the Lagos State University Teaching Hospital between 1^st^ October and 31^st^ December 2011. The study protocol was approved by the Hospital Research and Ethics Committee in LASUTH. Continuity corrected sample size was determined to be 304 subjects (152 apiece) using WINPEPI (PEPI-for-Windows) version 5.5: computer programs for epidemiological studies [18] at an estimated CS prevalence rate of 25% with power set at 80% and confidence level at 95%. Consecutive parturients with normal singleton pregnancies who had caesarean section and the immediate next parturients who had vaginal delivery and consented to participate in the study on the same day were recruited as study group and control respectively. Indications for caesarean section were as determined by the surgeons in each case. Data was collected using a pre-tested proforma.

### Variables

The maternal variables studied included socio-demographic, and anthropometric factors such as age, occupation, marital status, parity, booking status, weight, height. Obstetric parameters such as bleeding earlier in the index pregnancy and presence of previous caesarean section were also sought. The neonatal variable studied was birth weight.

### Data processing and analysis

Data obtained from the proforma were analysed using SPSS 16.0 Windows Evaluation version, Chicago, USA. Descriptive statistics (minimum, maximum, mean, and standard deviation) were calculated for continuous variables. Percentages and proportions were determined for categorical variables. Binary logistic regression model was estimated using the Entry method. This was validated using the forward and backward LR method to check for consistency and was used to determine the predictive factors of caesarean section in the study.

Total cases, Hosmer-Lemeshow goodness-of-fit statistics, model chi-square, observed groups and predicted probabilities chart, residual chi-square would be utilized. For each variable in the equation: coefficient (B), standard error of B, Wald statistic, unadjusted odds ratio (exp (B), confidence interval for exp (B), adjusted odds ratio would be determined. Pearson’s Chi Square was used to assess the significance of relationship between categorical variables.

A p-value less than 0.05 was considered to be statistically significant.

## Results

During the 2 month study period, there were 641 deliveries out of which 257 were by caesarean section. The caesarean section rate was 40.1% from this study. 47 (29.4%) of them were elective while 113 (70.6%) were emergency caesarean sections. The decision to undergo caesarean section was made at the level of the registrar in 18.0%, senior registrar in 48% and 34% at the level of the consultant.

Table 1 illustrates the socio-demographic characteristics of the parturients. The mean age of the women who had CS was 30.92 ± 4.85 years while the mean age of the control group was 29.86 ± 4.86 years.

**Table 1:** Socio-demographic factors

**Characteristics**	**Caesarean section**	**Spontaneous vaginal delivery**	**Total**	**p-value**
	**n (%)**	**n (%)**		
**Age (years)**				
< 20	4 (2.5)	0 (0)	4	
20-25	11 (6.9)	19 (11.9)	30	
26-30	61 (38.1)	82 (51.2)	143	0.613
31-35	62 (38.8)	41 (25.6)	103	
>35	22 (13.8)	18 (11.2)	40	
Total	160 (100)	160 (100)	320	
Occupation*				
Skilled worker	31 (21.5)	52 (32.5)	83	
Semi-skilled worker	8 (5.6)	10 (6.2)	18	
Unskilled worker	84 (58.3)	77 (48.1)	161	0.171
Unemployed	21 (14.6)	21 (13.1)	42	
Total	144 (100)	160 (100)	304	
**Marital status**				
Married	144 (90.0)	147 (91.9)	291	
Single	13 (8.1)	9 (5.6)	22	
Divorced	2 (1.3)	4 (2.5)	6	0.509
Widowed	1 (0.6)	0 (0)	1	
Total	160 (100)	160 (100)	320	
**Parity**				
Nulliparous	61 (38.1)	59 (37.1)	120	
Multiparous	99 (61.9)	100 (62.9)	199	0.851
Total	160 (100)	159 (100)	319	
**Booking status**				
Booked	130 (81.2)	128 (81.5)	258	
Unbooked	30 (18.8)	29 (18.5)	59	0.949
Total	160 (100)	157 (100)	317	

*Skilled workers: teachers, lawyers, journalists, accountants, secretary. Semi-skilled workers: tailors, hairdressers, caterers, typists; Unskilled workers: traders, cleaners, farmers, hospital maids, labourers; unemployed: students, full housewife, applicants.

Booking status of patients was not significantly associated with caesarean section at 81.2% and 81.5% respectively.

Most of the cases and controls were multiparous (61.9% and 62.9% respectively). Only 38.1% and 37.1% respectively were nulliparous. p = 0.851. Parity was not significantly associated with caesarean section.

The anthropometric characteristics of the parturients are depicted in Table 2. The height of the women who had CS ranged from 149 - 171 cm (mean 158.5 ± 4.7 cm) while that of the controls ranged from 148 – 173 cm (mean 159.3 ± 4.8 cm). The caesarean section rate was highest (60.0%) among women who were less than 151 cm in height and least among those 171 - 180 cm in height (16.7%). Height of women was not significantly associated with caesarean section {p = 0.466}.

**Table 2:** Anthropometric factors

**Height (cm)**	**Caesarean section**	**Spontaneous vaginal delivery**	**Total**	**p-value**
	**n (%)**	**n (%)**		
<151	6 (60.0)	4 (40.0)	10	
151-160	109 (51.2)	104 (48.8)	213	
161-170	44 (48.9)	46 (51.1)	90	0.358
171-180	1 (16.7)	5 (83.3)	6	
Total	160	159	319	
**Weight (kg)**	**Caesarean section**	**Spontaneous vaginal delivery**	**Total**	**p-value**
	**n (%)**	**n (%)**		
50-69	28 (31.0)	43 (69.0)	71	
70-89	59 (44.8)	78 (55.2)	137	< 0.001
≥ 90	38 (73.5)	12 (26.5)	50	
Total	125	133	258	

The weights of the cases ranged from 55 - 125 kg (mean 85.6 ± 13 kg) while those of the controls were between 56 - 113 kg (mean 77.5 ± 10.8 kg). The caesarean section rate was highest (73.5%) among women who weighed 90 kg and was lowest amongst those who weighed 50 - 69 kg (31.0%). Maternal weight was found to be significantly associated with caesarean section (p < 0.001).

Table 3 shows birth weights in both groups. Among the cases, the birth weight range was 1.40 - 4.40 kg (mean 3.18 ± 0.62 kg) while in the controls it was 1.05 - 4.20 kg (mean 3.09 ± 0.53 kg). The highest caesarean section rate (80%) was in women with fetal weights of 1.60 - 2.49 kg while the least was in the 2.50 – 3.99 kg (45.7%) group. Babies with birth weight of 4.0 kg and above had caesarean section rate of 66.7%. The birth weight of the babies was found to be significantly associated with caesarean section p < 0.001.

**Table 3:** Birth weight

**Weight (kg)**	**Caesarean section**	**Spontaneous vaginal delivery**	**Total**	**p-value**
	**n (%)**	**n (%)**		
1.01 – 1.59	3 (50.0)	3 (50.0)	6	
1.60 – 2.49	20 (80.0)	5 (20.0)	25	
2.50 – 3.99	118 (45.7)	140 (54.3)	258	0.005
4.00 – 5.00	12 (66.7)	6 (33.3)	18	
Total	153	154	307	

The role of factors in obstetric history is highlighted in Table 4. The rate of caesarean section was 86.7% among those patients who had a history of bleeding in the pregnancy, while it was 48.2% among those who did not. History of antepartum bleeding was found to be significantly associated with caesarean section (p = 0.006). The caesarean section rate was 93.0% among women who had previous caesarean section and it was 34.2% among those who did not. Previous caesarean section was also found to be significantly associated with caesarean section (p < 0.001).

**Table 4:** Obstetric factors

**Obstetric factors**	**Caesarean section**	**Spontaneous vaginal delivery**	**Total**	**p-value**
	**n (%)**	**n (%)**		
**History of vaginal bleeding**				
Yes	13 (86.7)	2 (13.3)	15	
No	147 (48.2)	158 (51.8)	305	0.006
Total	160	160	320	
**Previous caesarean section**				
Yes	80 (93.0)	6 (7.0)	86	
No	80 (34.2)	154 (65.8)	234	< 0.001
Total	160	160	320	

The logistic regression coefficient for predicting the mode of delivery in Tables 5 &6 showed that the parity, maternal height, maternal weight, birth weight, previous caesarean section and ante-partum bleeding were significant risk factors for caesarean section. Only the maternal age was not a significant risk factor for caesarean section (OR = 0.99, 95% CI = 0.3 – 2.8, p = 0.979).

**Table 5:** **Crude odds ratio of predictive factors from** bivariate analysis

**Predictive factor**	**OR**	**CI of OR**	**P-value**
Weight at delivery (≥ 90 Kg)	3.78	2.18 – 6.57	< 0.001
Birth Weight (≥ 2.5 Kg)	0.31	0.13 – 0.72	0.006
Birth Weight (≥ 4 Kg)	2.10	0.77 – 5.75	0.149**
Apgar score at 5 min (1 - 6)	2.77	1.12 – 6.84	0.027
Previous Caesarean section	25.67	10.73 – 61.42	0.00
APH	6.99	1.55 – 31.49	0.01
Deformity of Pelvis	-	-	-

Note: Confedence Interval (CI) of OR is at 95% confidence level.

** = Odds ratio (OR) not significant at 95% confidence level.

**Table 6:** Logistic regression identifying predictive factors of caesarean section

	**B - coefficient**	**Standard error**	**Odds ratio**	**95% CI of OR**	**p-value**
Age of mother	-0.014	0.541	0.986	0.342 - 2.845	.979
Parity	1.458	0.346	4.297	2.181 - 8.464	.000
Height of mother	0.909	0.370	2.482	1.202 - 5.124	.014
Maternal Weight	-1.113	0.269	0.329	0.194 - 0.557	.000
Birth weight	-1.357	0.536	0.257	0.090 - 0.735	.011
Previous Caesarean section	-3.654	0.491	0.026	0.010 - 0.068	.000
Antepartum Bleeding	2.075	0.929	7.968	1.290 - 49.213	.025
Constant	-2.077	1.263	0.125		.000

CI = confidence interval, OR = Odds ratio.

The various indications for caesarean section are listed on Table 7. The commonest indications for Caesarean section were failure to progress CPD/Obstructed labour (19.3%), eclampsia (11.3%), malpresentation/malposition (13.1%), fetal distress (8.1%) and antepartum haemorrhage (8.1%).

**Table 7:** Indications for caesarean section

**Indications**	**Caesarean section**	**Percentage**
Failure to Progress (CPD + Obstructed Labour)	31	19.3
Eclampsia (+Severe Pre-eclampsia)	18	11.3
Fetal Malpresentation/Malposition	21	13.1
Fetal distress	13	8.1
Antepartum haemorrhage	13	8.1
Previous C/S ≥ 2 x	13	8.1
Fetal Macrosomia	10	6.3
Previous Myomectomy	8	5.0
Failed induction	6	3.8
Previous C/S + Postdatism/Previous C/S + Inadequate Pelvis	12	7.5
PMTCT	6	3.8
Uterine Fibroid	4	2.5
Older Primigravida	2	1.3
Bad Obstetric History	2	1.3
Short Stature	1	0.6
**Total**	**160**	**100.0**

## Discussion

The Caesarean section rate in this study was 40.1%. This is higher than the rate of 22% [19] and 34.7% [20] found in earlier studies in tertiary hospitals in South-west Nigeria. It is also much higher than the 15% recommended by World Health Organisation [21] but comparable to 43% rate from public hospitals in Brazil [22]. The emergency caesarean section rate is much higher (70.4%) than the elective caesarean section (29.4%). This result is similar to earlier studies in developing countries [23,24] and might be because of the prevalence of such factors as cephalo-pelvic disproportion and prolonged obstructed labour which are diagnosed in labour. Another probable explanation could be the great aversion to operative delivery in this environment which makes women ‘surrender’ to surgery as a last result [23]. The socio-demographic characteristics in this study showed the marital status and occupation of patients are similar in both study population. Maternal age was not found to be a significant risk factor in this study (OR = 0.986, p = 0.979) in contrast to other studies that showed that increase in maternal age is associated with increase in caesarean section rates [19,25]. This may be due to the relatively younger age distribution of the patients in this study where most of the patients were aged between 26-35 years with only few patients above 35 years compared to other studies [14].

There were few more unbooked patients who were delivered by caesarean section compared to those who had vaginal delivery (18.8% versus 18.5%). This may be because they were complicated cases referred from private hospitals and traditional homes. This is similar to reports from other studies in Nigeria and Africa [14,19], and a probable contributor to the high caesarean section rate.

In this study patients with low parity were more likely to have caesarean delivery than those with higher parity. The caesarean section rate was highest among patients with low parity (para 1 or 2). Although univariate analysis of parity alone was not significantly associated with caesarean section but multivariate analysis using the logistic regression coefficient showed parity was a statistically significant predictive factor for caesarean section. This is similar to the results in earlier studies [1,14].

This study showed that increase in maternal weight was associated with increased risk of caesarean section (OR = 0.329, p < 0.01). This may be because increase in maternal weight and body mass index is associated with increased risk of fetal macrosomia leading to increased risk of cephalopelvic disproportion and need for caesarean delivery and is similar to findings from other studies [26,27].

In this study, univariate analysis of the maternal height alone was not found to be significantly associated with caesarean section. However, multiple regression analysis showed that maternal height is a significant predictive factor for caesarean section (OR = 2.482, p = 0.014). A short maternal stature is associated with an increased risk of obstructed labour due to cephalopelvic disproportion and most antenatal programmes designate short women as at risk. Other studies have also found height to be a predictive factor for caesarean section [28,29].

Increase in birth weight was associated with an increased caesarean section risk in this study. The difference was more pronounced at birth weights between 1.6 and 2.5 kg (80% to 20%) as well as 4 kg and above (66.7% to 33.3%). Fetal macrosomia is associated with increased risk of cephalopelvic disproportion leading to delivery by caesarean section [14,19,28].

Previous caesarean section was found to be statistically significant as a predictive factor for caesarean section in this study at 93% to 7%. There is a lower threshold for caesarean section after a previous caesarean section and this has contributed to the rise in caesarean section rate all over the world [30,31] In conclusion, the logistic regression analysis showed that maternal age was not a significant predictor of caesarean section at Lagos State University Teaching Hospital, Ikeja. However, maternal weight, height, parity, previous caesarean section, antepartum haemorrhage and birth weight were significant predictive factors. Odds ratio showed that maternal weight, birth weight, and previous caesarean section were strong causal predictors of caesarean section (OR = 0 – 0.3). Hence, women with weight above 90 kg, fetal birth weight ≥ 4.0 kg and women with history of caesarean section are more likely to deliver by caesarean section. Parity, maternal height and antepartum bleeding were identified by odds ratio as protective predictive factors of caesarean section. Multiparous women with height greater than 1.60 m and without antepartum bleeding were less likely to undergo caesarean section (OR > 2.6). The commonest indications for caesarean section in this study were failure to progress (often due to cephalopelvic disproportion), eclampsia (including severe pre-eclampsia), breech presentation, fetal distress and antepartum haemorrhage. These form 55.6% of all the indications for caesarean section and are similar to findings from other studies [9,32,33]. Most of these indications are predictable and when detected should alert caregivers to counsel pregnant women and refer them to centers where caesarean delivery would be offered. While every effort is justifiable, to keep caesarean section rate reasonably low, both maternal and fetal morbidity have been reported to be higher in centers where caesarean section rates are lower than 10% [2,11] as this may be a reflection of suboptimal care. A limitation of this study was its timing, which coincided with a period when it was announced that the hospital was winding down its activities in preparation for a massive renovation,and referrals to satellite hospitals were actively encouraged. This might have affected the number of unbooked patients seen … Another limitation was the non inclusion of gestational age in the variables considered. However, direct correlation between birth weight and gestational age [34] at the same centre could provide a useful guide for inferences.

## Conclusions

This study has demonstrated that increasing maternal weight ≥ 4 kg, short stature, low parity, history of previous caesarean section, ante partum haemorrhage as well as estimated birth weight between 1.6 kg and 2.5 kg and birth weight above 4 kg are associated with increased risk of caesarean section. These women should therefore be counselled early at presentation to book at centres equipped for surgery.

## Competing interests

The authors declare that they have no competing interest.

## Authors’ contributions

AOI, responsible for the conception, design and acquisition of data for this study and drafting and revising the manuscript. TAO, responsible for the conception, design. AEM, Involved in design and acquisition of data for this study. FAO, Responsible for conception, drafting and revising the manuscript for publication. KAR, Responsible for interpretation of data and revising the manuscript for intellectual content. OYA, involved in interpretation of data and drafting of the manuscript. All authors read and approved the final manuscript.

## Pre-publication history

The pre-publication history for this paper can be accessed here:

http://www.biomedcentral.com/1471-2393/14/217/prepub
